# PPARβ/δ activation of CD300a controls intestinal immunity

**DOI:** 10.1038/srep05412

**Published:** 2014-06-24

**Authors:** Toshiya Tanaka, Satoko Tahara-Hanaoka, Tsukasa Nabekura, Kaori Ikeda, Shuying Jiang, Shuichi Tsutsumi, Takeshi Inagaki, Kenta Magoori, Takuma Higurashi, Hirokazu Takahashi, Keisuke Tachibana, Yuya Tsurutani, Sana Raza, Motonobu Anai, Takashi Minami, Youichiro Wada, Koutaro Yokote, Takefumi Doi, Takao Hamakubo, Johan Auwerx, Frank J. Gonzalez, Atsushi Nakajima, Hiroyuki Aburatani, Makoto Naito, Akira Shibuya, Tatsuhiko Kodama, Juro Sakai

**Affiliations:** 1 Laboratory for Systems Biology and Medicine (LSBM), Research Center for Advanced Science and Technology (RCAST), The University of Tokyo, Tokyo 153-8904, Japan; 2 Department of Immunology, Faculty of Medicine, Center for TARA and Japan Science and Technology Agency, CREST, University of Tsukuba, Tsukuba 305-8575, Japan; 3 Division of Cellular and Molecular Pathology, Niigata University Graduate School of Medical and Dental Sciences, Niigata 951-8510, Japan; 4 Peruseus Proteomics, Tokyo 153-0041, Japan; 5 Genome Science Division, Research Center for Advanced Science and Technology (RCAST), The University of Tokyo, Tokyo 153-8904, Japan; 6 Division of Metabolic Medicine, Research Center for Advanced Science and Technology (RCAST), The University of Tokyo, Tokyo 153-8904, Japan; 7 Gastroenterology Division, Yokohama City University School of Medicine, Yokohama 236-0004, Japan; 8 Graduate School of Pharmaceutical Sciences, Osaka University, Osaka 565-0871, Japan; 9 Department of Clinical Cell Biology and Medicine, Chiba University Graduate School of Medicine, Chiba 260-8670, Japan; 10 Laboratory for Vascular Biology, Research Center for Advanced Science and Technology (RCAST), The University of Tokyo, Tokyo 153-8904, Japan; 11 Department of Quantitative Biology and Medicine, Research Center for Advanced Science and Technology (RCAST), The University of Tokyo, Tokyo 153-8904, Japan; 12 Laboratory of Integrative and Systems Physiology, Ecole Polytechnique Fédérale de Lausanne, 1015 Lausanne, Switzerland; 13 Laboratory of Metabolism, Center for Cancer Research, National Cancer Institute, National Institutes of Health, Bethesda, MD 20892

## Abstract

Macrophages are important for maintaining intestinal immune homeostasis. Here, we show that PPARβ/δ (peroxisome proliferator-activated receptor β/δ) directly regulates *CD300a* in macrophages that express the immunoreceptor tyrosine based-inhibitory motif (ITIM)-containing receptor. In mice lacking CD300a, high-fat diet (HFD) causes chronic intestinal inflammation with low numbers of intestinal lymph capillaries and dramatically expanded mesenteric lymph nodes. As a result, these mice exhibit triglyceride malabsorption and reduced body weight gain on HFD. Peritoneal macrophages from *Cd300a−/−* mice on HFD are classically M1 activated. Activation of toll-like receptor 4 (TLR4)/MyD88 signaling by lipopolysaccharide (LPS) results in prolonged IL-6 secretion in *Cd300a−/−* macrophages. Bone marrow transplantation confirmed that the phenotype originates from CD300a deficiency in leucocytes. These results identify CD300a-mediated inhibitory signaling in macrophages as a critical regulator of intestinal immune homeostasis.

The intestinal tract is the largest surface organ of the human body which is constantly exposed to dietary and environmental antigens such as commensal bacteria. Therefore, the intestinal immune system has to maintain homeostasis through the cooperation of various regulatory mechanisms that prevent overreaction against beneficial flora and food antigens^1^,^2^. Dysregulation of intestinal immune responses is believed to cause inflammatory bowel diseases (IBD), Crohn's disease and ulcerative colitis, metabolic diseases such as obesity and diabetes, and is also linked to autoimmune disease^3^,^4^. The largest number of macrophages in the body reside in intestine and these cells are important for maintaining intestinal immune homeostasis^5^. This function is controlled by positive and negative signals by activating and inhibitory cell surface immune receptors, respectively^6^. In fact, lack of cell surface immune receptor Trem2, known to inhibit Myd88-mediated TLR signaling, has been shown to increase in proinflammatory M1 marker cytokine production in macrophage and impaired wound healing^7^.

The nuclear receptor PPARβ/δ has been shown to transcriptionally-regulate oxidative metabolism in muscle and improve insulin sensitivity^8^,^9^. In macrophages, PPARβ/δ modulates resident macrophage polarization through the Th2 cytokine signaling cascade^10^ and reduces atherogenic inflammation^8^. It has been indicated that PPARβ/δ agonist represses inflammatory gene expression by releasing transcriptional co-repressor BCL-6 in macrophages^11^. In addition, PPARβ/δ has been supposed to attenuate chemokine receptor signaling by the induction of RGS proteins, which is involved in the termination of G protein signal^12^,^13^. However, the mechanisms underpinning anti-inflammatory properties of PPARβ/δ have not been fully understood.

In the current study, we performed comprehensive analysis of PPARβ/δ regulated genes and genome-wide PPARβ/δ binding sites to facilitate our understanding of the PPARβ/δ function in macrophages. We found that lack of CD300a, a novel PPARβ/δ target gene, expression in leucocytes relieves the TLR4/Myd88 signaling which leads to increase in proinflammatory cytokines in macrophages.

## Results

### PPARδ activates *Cd300a* in macrophages

**Figure 1:**
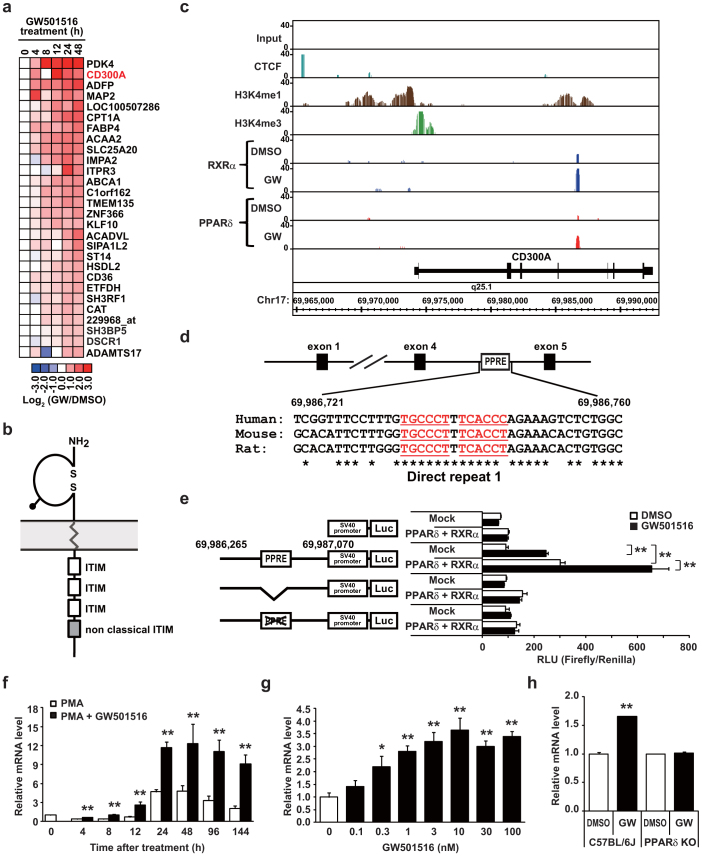
*Cd300a* is a direct target of PPARδ. (a) Heat map: Color denotes the GW501516-induced changes in THP-1 macrophages. (b) Schematic diagram of CD300a. (c) Histogram of ChIP fragments. (d,e) PPRE present in intron 4 of *Cd300a* gene is conserved among species (d) and can mediate *Cd300a* induction by PPARβ/δ (e). Error bars show s.e.m. **P < 0.01. (f,g) Time course of *Cd300a* expression in THP-1 cells exposed to GW501516/PMA (f) and dose response in THP-1 macrophages treated with GW501516 (g). (h) *Cd300a* induction by GW501516 is blunted in peritoneal macrophages from PPARβ/δ-null mice (n = 3). Error bars show s.e.m. *P < 0.05; **P < 0.01 compared with DMSO treatment.

To investigate the function of PPARβ/δ (NR1C2) in macrophages, we treated THP-1 macrophages with the high-affinity PPARβ/δ agonist GW501516 and performed a time course of global gene-expression analyses. These were combined with ChIP-seq analyses using newly-generated monoclonal antibodies against PPARβ/δ, as well as antibodies against its heterodimer partner, RXRα^14^ (Supplementary Fig. S1a–e). We also generated genome-wide maps of modification sites for histone H3 lysine 4 mono- and tri-methylation (H3K4me1 and me3, respectively). Additionally, we identified binding sites for the insulator binding protein, CCCTC-binding factor (CTCF). GW501516 treatment induces the expression of 34 genes and ChIP-seq analyses identified 28 of these genes as direct targets of PPARβ/δ (Fig. 1a). These include known PPARβ/δ targets whose gene products are involved in fatty acid metabolism, such as *Pdk4*, *Cpt1a*, and *Slc25A20* (Supplementary Fig. S1f). Interestingly, PPARβ/δ also directly regulates genes encoding molecules that potentially inhibit signaling of the immunoreceptor tyrosine-based activation motif (ITAM) (*Cd300a*, *Sh3bp5*, and *Dscr1*) (Fig. 1a). In fact, *Cd300a* was one of the most robustly induced genes.

CD300a is an inhibitory immunoreceptor that fine tunes innate immune cell activity through an ITIM-mediated inhibitory signal (Fig. 1b)^15^. It is preferentially expressed on cell surface of myeloid-lineage cells including macrophages, dendritic cells and mast cells^16^. In macrophages^17^,^18^ and in mast cells^18^, CD300a inhibits the TLR4 (a receptor for LPS and fatty acids) signaling pathway, leading to the inhibition of the innate immune system^19^. However, the physiological function of CD300a in macrophages is not fully understood yet.

ChIP-seq analysis revealed the binding of PPARβ/δ and RXRα to sites downstream of exon 4 in *Cd300a*. These sites were flanked by H3K4me1 modifications (Fig. 1c, Supplementary Fig. S2a). Because H3K4me1 is a marker for histone chromatin modification of enhancer regions, these results indicate that *Cd300a* gene expression is regulated directly by the PPARδ/RXRα heterodimer. In agreement with this result, we identified a potential PPAR responsive element (TGCCCT T TCACCT/C; PPRE) in this intron (Fig. 1d). This PPRE is conserved among mouse, rat, and human and was able to mediate PPARβ/δ dependent transactivation. Cotransfection of the luciferase reporter vector with a PPARβ/δ and RXRα expression vector increased luciferase activity, and this was further enhanced by the addition of GW501516 (Fig. 1e). Moreover, GW501516 treatment consistently increased the abundance of *Cd300a* mRNA in THP-1 macrophages in a time- and dose-dependent manner (Fig. 1f,g). This induction was abrogated by shRNA-mediated knock down of PPARβ/δ (Supplementary Fig. S2b,c). GW501516-mediated *Cd300a* gene induction was observed in peritoneal macrophages from wild-type mice but not from PPARβ/δ-null mice (Fig. 1h). Another PPARβ/δ specific agonist, L-165041, also induced *Cd300a* mRNA (Supplementary Fig. S2d). Taken together these data identify *Cd300a* as a bona fide target of PPARβ/δ.

### *Cd300a* deficiency causes gut inflammation under HFD feeding

**Figure 2:**
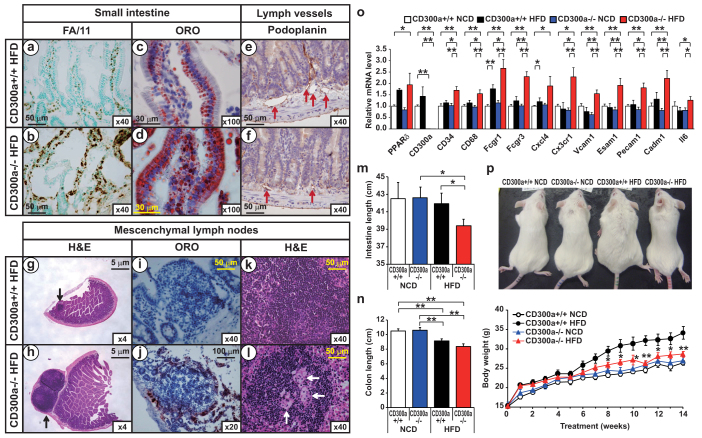
*Cd300a* deficiency causes gut inflammation under HFD feeding. (a–l) Jejunal cross sections from 18-week-old HFD-fed littermates were stained with antibodies for CD68 (FA/11) (a, b) or podoplanin (e, f) or with oil red O (ORO) (c, d, i, j) or haematoxylin-and-eosin (H&E) (g, h, k, l). Red, black, and white arrows denote lymphcapillaries, lymphnodes, and foam cells, respectively. (m) Small intestinal length (n = 5–6 per group). (n) Colonic length (n = 5–6 per group). (o) Expressions of pro-inflammatory cytokines and chemokines in the small intestine of littermates (n = 4 mice per group). Error bars show s.e.m. *P < 0.05; **P < 0.01. (p) Body weight changes of female littermates on BALB/c genetic background mice fed on HFD from 4 weeks old (n = 5–6 per group). Photograph shown is representative 18-week-old littermates. *P < 0.05; **P < 0.01 compared with *Cd300a+/+* mice on HFD.

To gain insights into the biological role of CD300a, we generated CD300a-null mice as described elsewhere^18^. We fed these animals a diet rich in lard-based SFAs (HFD) (Supplementary Table S1a). Histological examination revealed enhanced immune staining of FA/11^+^(CD68) and F4/80^+^ macrophages and Thy1.2^+^ T cells in the intestine and colon of *Cd300a−/−* mice fed HFD (Fig. 2a,b, Supplementary Fig. S3). Oil red O (ORO) staining revealed marked lipid accumulation in the intestinal epithelium of these mice (Fig. 2c,d). Lymphatic capillaries were very narrow and hardly visible in the intestine from these *Cd300a−/−* mice fed HFD (Fig. 2e,f). Furthermore, the mesenteric lymph nodes (MLNs) were markedly expanded with accumulation of lipid-laden macrophages and foam cell formation indicating inflammation in MLNs (Fig. 2g–l). In addition, the length of small intestine and colon of *Cd300a−/−* mice are shortened by HFD feeding (Fig. 2m,n). Transcriptome analyses of intestine showed up-regulation of the expression of chemokine and pro-inflammatory genes (*FCγr1*, *FCγr3, Cxcl4, Cx3cr1, and Il6*), adhesion molecules (*Vcam1, Esam1*, and *Pecam1*) and the macrophage marker *Cd68* in the same 18-week-old *Cd300a−/−* mice on HFD. These data suggest that macrophages are responsible for this chronic intestinal inflammation (Fig. 2o).

### *Cd300a−/−* mice shows impaired lipid absorption under HFD feeding

**Figure 3:**
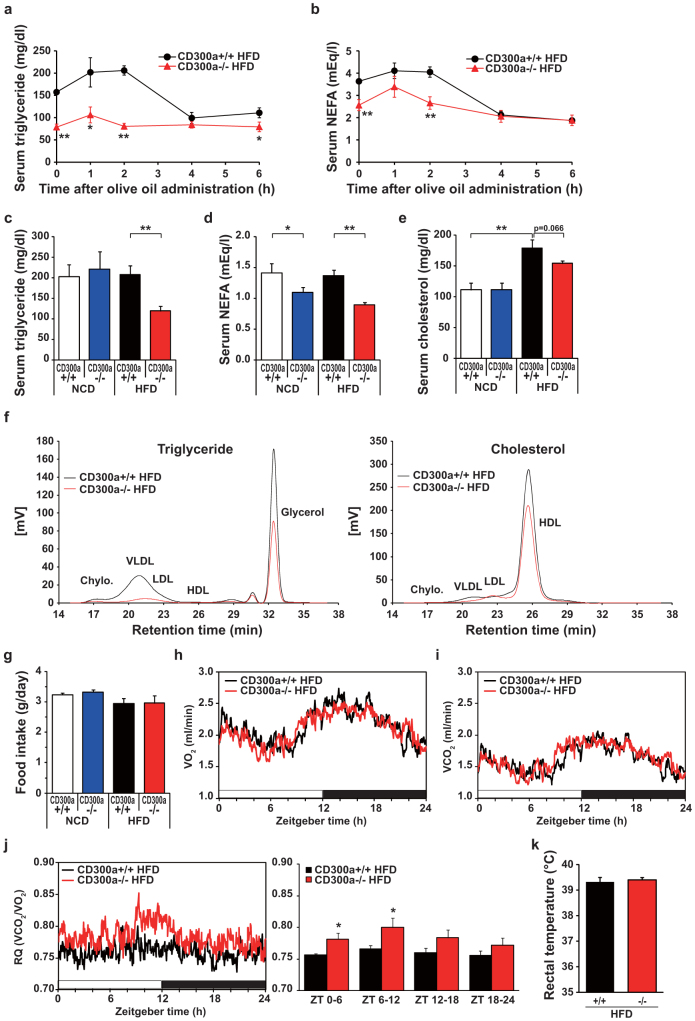
*Cd300a* deficiency causes triglyceride malabsorption under HFD feeding. (a,b) Acute fat loading test (*Cd300a+/+*, *n* = 4; *Cd300a−/−*, n = 5). (c–e) Serum TG, NEFA, and cholesterol concentrations (n = 5–6 per group). (f) HPLC analysis of serum lipoproteins. Chylo. chylomicron; VLDL, very low-density lipoprotein; LDL, low-density lipoprotein; HDL, high-density lipoprotein. (g) Food intake. (h) Oxygen consumption [VO_2_], (i) CO_2_ production rate [VCO_2_], and (j) Respiratory quotient (RQ) (left) of *Cd300a+/+* and *Cd300a−/−* mice (11–15 weeks old, n = 5 per group) fed on HFD used in Fig. 2. Note that RQ was significantly higher in *Cd300a−/−* mice during light cycle (i.e. Fasting phase) indicating fat utilization is lower in *Cd300a−/−* mice during fasting phase. (k) Rectal temperature at 18 weeks of age (n = 6 per group). Data represent the mean ± s.e.m. *P < 0.05; **P < 0.01.

Since dietary lipids are absorbed from the intestine by the lymph capillaries, we next examined the fat absorption of HFD fed *Cd300a−/−* mice. Acute fat-loading demonstrated that triglyceride (TG) levels in *Cd300a−/−* mice remained low in contrast to the *Cd300a+/+* mice, and no peak in serum TG levels at 2 h occurred (Fig. 3a,b). Consistently, serum lipid content, including non-esterified free fatty acid (NEFA) and TG, is significantly reduced in the same *Cd300a−/−* mice on HFD (Fig. 3c–e). HPLC analysis of plasma lipoproteins showed a substantial reduction in chylomicron and very low-density lipoprotein (VLDL) TG levels in HFD-fed *Cd300a−/−* mice (Fig. 3f). Food intake, oxygen consumption, and rectal temperature showed no significant differences between *Cd300a+/+* and *Cd300a−/−* mice (Fig. 3g–k). RQ value is significantly higher in HFD fed *Cd300a−/−* mice than that of *Cd300a+/+* (*P < 0.05*) indicating lower utilization of lipid as an energy source (Fig. 3j). These observations indicated that the chronic intestinal inflammation associated with lymph capillary obstructions led to impaired intestinal lipid absorption of HFD fed *Cd300a−/−* mice. As a result of impaired lipid absorption, *Cd300a−/−* mice gained less body weight relative to *Cd300a+/+* mice on HFD (Fig. 2p, Supplementary Fig. S4a–e). Feeding palm oil-based high saturated fat diets resulted in a similar phenotype to the lard-based HFD (Supplementary Table S1b, Supplementary Fig. S5a–c).

### Hematopoietic deficiency of CD300a augments TLR4 mediated IL6 production and exacerbates intestinal inflammation

**Figure 4:**
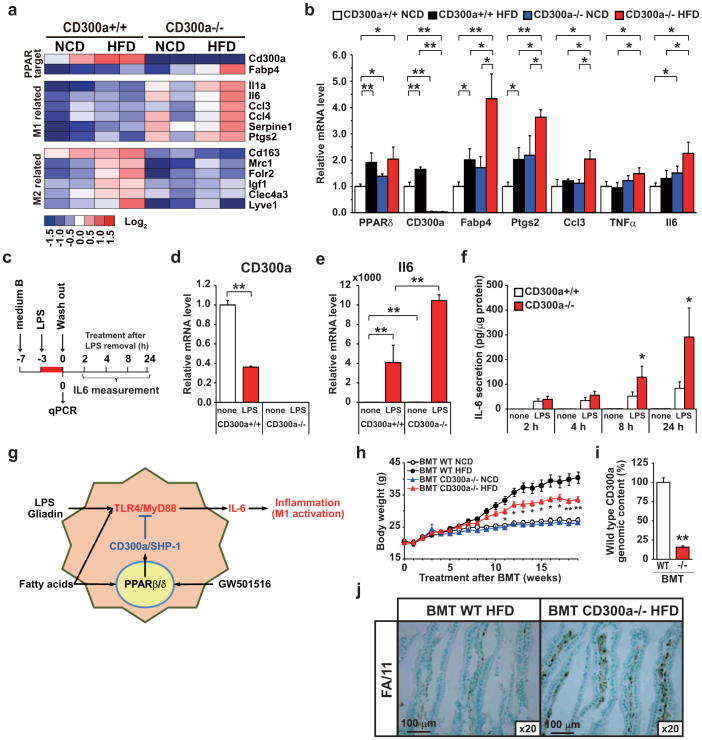
Hematopoietic deficiency of CD300a exacerbates intestinal inflammation and impairs body weight gain. (a) Heat map showing changes in expression of selected M1 and M2 markers in NCD- or HFD-fed *Cd300a+/+* or *Cd300a−/−* mouse peritoneal macrophages. (b) qPCR validation of M1 pro-inflammatory markers (n = 3). Error bars show s.e.m. *P < 0.05; **P < 0.01. (c–f) Peritoneal macrophages were cultured and treated with LPS (200 ng/ml) for 3 h, washed, and subsequently cultured in LPS-minus medium. Cells were harvested at the indicated time points (c). qPCR expressions (d, e) and IL-6 release (f) were determined. Error bars show s.e.m. *P < 0.05; **P < 0.01. (g) Model depicting the role of the PPARβ/δ-CD300a axis inhibits TLR4/MyD88 pathway in macrophage. Fatty acids also serve as ligands to activate PPARβ/δ to control the expression of *Cd300a* as well as genes encoding oxidative metabolism. (h) Body weight changes of C57BL/6J littermates reconstituted with bone marrow of either wild-type or *Cd300a−/−* C57BL/6J mice (BMT-WT and BMT-*Cd300a−/−*, respectively) (n = 4–6 per group). *P < 0.05; **P < 0.01 compared with BMT-*Cd300a*+/+ mice on HFD. (i) Replacement of wild-type bone marrow by *Cd300a−/−* bone marrow cells (n = 10 and 11, respectively). (j) CD68(FA/11) staining of jejunal cross sections. Error bars show s.e.m. **P < 0.01 compared with BMT-*Cd300a*+/+ mice.

To examine the influence of CD300a deficiency on macrophages, we used oligonucleotide microarray and qPCR to examine classically-activated pro-inflammatory M1 and alternatively-activated anti-inflammatory M2 macrophage marker gene expressions in peritoneal macrophages. This analysis revealed that macrophages from *Cd300a+/+* mice on HFD resemble alternatively-activated anti-inflammatory M2 macrophages. They express a number of M2 markers including *Cd163, Mrc1, Folr2, Igf1, Clec4a3*^20^,^21^,^22^, whereas those from *Cd300a−/−* mice expressed classically-activated M1 markers including *Il1a, Il6, Ccl3, Ccl4, Serpine1, Ptgs2, Tnfa*^20^,^23^ (Fig. 4a,b, Supplementary Fig. S6). *Lyve-1*^24^, is considered another M2-macrophage related gene^21^,^25^ whose product is pivotal in lymphatic vessel development. Intriguingly, *Lyve-1* was highly induced upon HFD in *Cd300a+/+* mice and this was not observed in *Cd300a−/−* mice (Fig. 4a,b). These data suggested that *Cd300a−/−* mice may have a defect in preventing the appropriate responses seen in chronic inflammatory and autoimmune diseases.

Because HFD induces activation of TLR4 signaling pathway and causes low-grade intestinal inflammation^26^, we asked whether lack of CD300a expression relieves the inhibition of TLR4 signaling and in turn lead to an increase in pro inflammatory cytokine production in macrophages. To examine this hypothesis, we treated peritoneal macrophages from *Cd300a+/+* and *−/−* mice with LPS for 3 h to trigger the TLR4 signaling pathway^27^ and subsequently cultured them in LPS-minus media (Fig. 4c). LPS treatment led to a 60% reduction of *Cd300* expression (Fig. 4d), consistent with results previous reported in monocytes^28^, and induced *Il6* expression (Fig. 4e). Lack of *Cd300a* in macrophages resulted in 2.5-fold higher expression of *Il6* upon LPS induction (Fig. 4e). Furthermore, while IL-6 secretion from *Cd300a+/+* macrophages continued at similar levels during 24 h incubation after removal of LPS, IL-6 secretion from *Cd300a−/−* macrophages did not cease even after 24 h in the absence of LPS (Fig. 4f). These data together with our previous data^18^ indicate that CD300a is pivotal in preventing or terminating TLR4-triggered *Il6* expression and IL-6 secretion (Fig. 4g).

To specify whether HFD-induced intestinal inflammation in *Cd300a−/−* mice originates from malfunction of intestinal resident macrophages, we adoptively transferred CD300a-deficient bone marrow into lethally-irradiated wild-type mice (male C57BL/6J strain). After reconstitution for 4 weeks, age-matched cohorts were placed on HFD or NCD for 15 weeks (Fig. 4h). qPCR analysis confirmed >84% replacement of wild-type marrow by *Cd300a−/−* cells (Fig. 4i). Reconstitution of wild-type mice with *Cd300a−/−* bone marrow led to enhanced macrophage infiltration in the intestine of BMT-*Cd300a−/−* mice (Fig. 4j), resulting in resistance to body weight gain on HFD (Fig. 4h) with no apparent changes in food intake (Supplementary Fig. S7a). This is similar to results observed in *Cd300a−/−* mice on a BALB/c genetic background. Serum lipid content and the mass of fat depots in adipose tissues were reduced in BMT-*Cd300a−/−* mice compared to BMT-*Cd300a+/+* mice on HFD (Supplementary Fig. S7b–g). Conversely, reconstitution of *Cd300a−/−* mice with wild-type bone marrow significantly restored weight gain on HFD (Supplementary Fig. S8a–d).

## Discussion

The small intestine is the organ that digests and absorbs dietary nutrients such as lipids. It is also exposed to a host antigens from the diet and from commensal bacterial. Fatty acids are a constituent of lipid nutrients and also serve as signaling molecules that influence biological processes. Fatty acids are also part of the lipid moiety of LPS and play an important role in activation of TLR4. Fatty acids thereby induce NF-κB target genes such as cyclooxygenase 2 (*Cox2* (*ptgs2*)), *Tnfa*, and *Il6* in macrophages^29^,^30^. By contrast, PPARβ/δ regulates expression of its target genes as a fatty acid sensor in macrophages^31^.

In the current study, we show that activation of PPARβ/δ induced the CD300a immunoreceptor. HFD feeding significantly induced intestinal inflammation in the *Cd300a−/−* and BMT-*Cd300a−/−* mice (Fig. 2,4). PPARβ/δ target *Cd300a* was induced in HFD fed *Cd300a+/+* mice macrophages (Fig. 2m,4b). In parallel, expression of M2 macrophage marker genes was also increased. By contrast, in macrophages of *Cd300a−/−* mice fed HFD, pro-inflammatory *Il6*, *ptgs2* (whose gene product is COX2), and *Tnfa* expression were increased (Figure 4a,b). These are all M1 macrophage markers. In cultured macrophages, LPS treatment led to the reduction of *Cd300a* expression (Fig. 4d). We also found that *Il6* expression and secretion by LPS pretreatment were enhanced in the *Cd300a−/−* macrophages (Fig. 4e,4f). Based on the previous literature, we postulate that this is mediated by the TLR4/Myd88 signaling pathway^17^,^18^. However, we do not exclude the possibility that other PPARβ/δ target molecules inhibit ITAM signaling (e.g. SH3BP5 and DSCR1) or that an anti-inflammatory co-repressor BCL-6^11^ suppresses inflammation, PPARβ/δ-CD300a axis suppressed inflammatory cytokine IL6 production induced by TLR4/Myd88 signaling by dietary antigens. Based on these finding we propose that CD300a, a novel PPARβ/δ target gene in macrophages, maintains intestinal immune response. In wild type mice, HFD-derived fatty acids can activate the TLR4/Myd88 pathway, but the PPARβ/δ-CD300a pathway inhibits TLR4/Myd88 pathway; therefore IL6 production is suppressed. In *Cd300a−/−* mice, HFD-derived fatty acids activate only the TLR4/Myd88 pathway and IL6 production proceeds as illustrated in Figure 4g.

Dysregulation of the intestinal innate immune response is linked to metabolic disease^32^ and type 1 diabetes (T1D)^3^. Interestingly, *Cd300a−/−* mice exhibit higher blood glucose levels with reduced serum insulin levels in *Cd300a−/−* mice and BMT-*Cd300a−/−* mice compared to controls (Supplementary Fig. S9a, S7h,i, respectively) despite of impaired lipid absorption. Glucose tolerance tests further showed that glucose-induced increases in serum insulin levels were significantly reduced in *Cd300a−/−* mice on HFD (Supplementary Fig. S9b). It has been demonstrated that pancreatic islet-infiltrating lymphocytes express α4β7-integrin, which is a homing receptor to the gut mucosa^33^. In addition, it is well known that endocrine and exocrine cells of the pancreas are derived from a common set of epithelial cells from early gut endoderm. These observation suggested that pancreatic β-cells are affected by the homing lymphocytes during intestinal inflammation. Therefore, the PPARβ/δ-CD300a axis may prevent food antigen-induced intestinal inflammation and metabolic diseases such as insulin resistance, atherosclerosis, and T1D.

These results suggest a novel mechanism through which PPARβ/δ activation leads to immuno-inhibitory receptor signaling to suppress chronic inflammation. The ability of PPARβ/δ to integrate metabolism and the innate immune system suggests that PPARβ/δ activation and subsequent CD300a induction could be a new therapeutic strategy to treat enteropathic diseases such as inflammatory-bowel-disease-like disease including Celiac disease^34^,^35^. In addition to our previous work which identified PPARβ/δ as a therapeutic target for the metabolic syndrome^9^, PPARβ/δ activation and its key role in intestinal immune modulation may also prove effective for the management of T1D.

## Methods

The methods used in this study are described in detail in Supplementary Information.

### Chemical reagents

Selective high affinity agonists of PPARβ/δ GW501516^36^ and fenofibric acid were synthesized as described previously^9^. L-165041 was purchased from Sigma-Aldrich, rosiglitazone (BRL 49653) from Cayman Chemical, phorbol 12-myristate 13-acetate (PMA) from Wako Pure Chemical Industries.

### Antibodies

Mouse monoclonal IgG-Y9705 against human PPARβ/δ and IgG-K8508 against human RXRα were raised in our laboratory by immunizing separate mice with recombinant baculovirus displaying gp64-fusion proteins containing amino acids 2–41 of human PPARβ/δ and 2–133 of human RXRα, respectively^37^. Other antibodies were obtained from the following sources: rabbit polyclonal anti-histone H3 trimethyl K4 (ab8580) from Abcam; polyclonal rabbit anti-CTCF (07-729) from Millipore; mouse monoclonal anti-Nucleoporin p62 (610497) and rat monoclonal anti-CD90.2 (550543, Thy-1.2) from BD Transduction laboratories; goat polyclonal anti-podoplanin (A-18) (sc-23564) from Santa Cruz biotechnology; rat monoclonal anti-CD68 (clone FA/11, MCA1957), anti-B220 (MCA1258G), and anti-F4/80 (MCA497GA) from AbD Serotec. Mouse monoclonal anti-histone H3 monomethyl K4 (clone CMA302) was a kind gift from Dr. H. Kimura.

### Histology

Haematoxylin and eosin (H&E) staining of sections was performed using standard protocols (Supplementary Information). For detection of macrophages, immunohistochemistry was performed using antibody against CD68 or F4/80 and, for detection of lymph vessels, antibody against podoplanin was used. For Oil red O staining, frozen sections embedded in OCT were air-dried and fixed in formalin followed by brief washing with water and rinsing with 60% isopropanol. Sections were immersed in freshly prepared Oil Red O working solution for 15 minutes followed by rinses with 60% isopropanol. Haematoxylin nuclei staining was performed subsequently.

### Cells

Human monocytic leukemia THP-1 cells were purchased from ATCC and maintained in RPMI 1640 medium (Invitrogen) supplemented with 10% heat-inactivated fetal bovine serum (FBS) containing 100 Uml^−1^ penicillin and 100 μg ml^−1^ streptomycin (Gibco) (medium A) at 37°C in 5% CO_2_. THP-1 monocytes were differentiated into macrophages with 10 nM PMA for 24 h as previously described. Raw 264.7 cells, a line of murine macrophage-like cells, were purchased from ATCC and maintained in DMEM containing 10% FBS, 100 Uml^−1^ penicillin and 100 μg ml^−1^ streptomycin (Gibco) at 37°C and 5% CO_2_.

Peritoneal macrophage cells from wild-type C57BL/6J (CLEA, Japan), CD300a-null, or PPARβ/δ-null mice^38^ were prepared as follows: mice were sacrificed and resident peritoneal cells were harvested by lavage with sterile phosphate buffered saline (PBS) (pH 7.4). After centrifugation at 190 × g for 5 min, cells were re-suspended in medium A and plated in 6-well plates and incubated for 2 h at 37°C in 5% CO_2_ to allow cells to attach to plates. Plates were subsequently washed with PBS to remove non-adherent cells. The resulting attached cells were used in experiments as peritoneal macrophages.

For LPS stimulation, macrophages cells were plated in 12-well plates in medium A on day 0 and, on day 1, cells were washed with PBS, cultured in RPMI1640 containing 0.1% FBS supplemented with 100 Uml^−1^ penicillin and 100 μg ml^−1^ streptomycin (Gibco) (medium B) for 4 h, then switched to medium B in the absence or presence of 200 ng/ml of LPS. After 3 h incubation, LPS were washed out with PBS 3 times, and cells were refed with medium B for the indicated period of time as described in the legend to Fig. 4c. IL-6 levels in the culture medium were determined by Mouse IL-6 Quantikine ELISA Kit (R & D systems) and normalized to total cellular protein levels.

### Transcriptome microarray analysis

For genome-wide transcription analysis, GeneChip Human Genome U133 Plus 2.0 Array or GeneChip Mouse Genome 430 2.0 array were used as described previously^9^,^14^ (Supplementary Information).

### ChIP-seq and ChIP-qPCR analysis

Chromatin immunoprecipitation (ChIP) was performed as described^14^ (Supplementary Information). Briefly, THP-1 cells after 24-h treatment with 10 nM PMA in the absence or presence of 100 nM GW501516 were cross-linked with 1% formaldehyde for 10 min at room temperature and chromatin DNA was sheared by sonication. The resultant was immunoprecipitated with the indicated antibodies overnight at 4°C^14^. After washing and elution, the protein-DNA complexes were reversed by heating at 65°C overnight. Immunoprecipitated DNA was purified by using QIAquick spin columns (Qiagen). ChIP sequencing (ChIP-seq) sample preparation was performed according to the manufacture**'**s instructions as described elsewhere (Ilumina)^14^. ChIP samples were also analyzed by gene-specific quantitative real time PCR.

### Quantitative real-time PCR (qPCR)

The qPCR method has been described^9^ (Supplementary Information). All primer sequences used in this paper are available on request.

### Lentiviral shRNA knockdown

To deplete cellular PPARβ/δ, MISSION Lentiviral Packaging Mix and the lentiviral shRNA transfer vectors (Sigma-Aldrich) were co-transfected into 293FT cells using Lipofectamine 2000 (Invitrogen). Lentiviral particles carrying shRNAs targeting human PPARβ/δ were used to infect THP-1 cells.

### Construction of the promoter reporter gene

pCD300a(806) is pGL3-Promoter-based luciferase reporter gene vector that harbors sequences from human CD300a intron 4 spanning positions from 69,986,265 to 69,987,070 of chromosome 17. To construct this plasmid, the corresponding region was amplified by PCR and cloned into pGL3-Promoter vector (Promega). Base substitution and deletion mutants were generated in pCD300a(806) with the QuikChange II site-directed mutagenesis kit (Stratagene).

### Luciferase reporter assay

Raw264.7 cells were transfected with the indicated reporter together with expression plasmids (pCMV-hPPARδ and pCMV-hRXRα)^9^ together with renilla luciferase plasmids (pRL-CMV) using Lipofectamine 2000 (Invitrogen) and treated with either with 100 nM GW501516 or vehicle (DMSO) 24 h after transfection^14^. After overnight treatment, cells were lysed in lysis buffer (Promega) and analyzed using the Dual-Luciferase® Reporter Assay System (Promega). Firefly luciferase signal was normalized to renilla luciferase signal. All luciferase assay data represent the mean ± s.e.m of triplicate samples.

### Animal experiments

*Cd300a*-*null* (BALB/cAJcl (BALB/c) back ground)^18^ and *PPARβ/δ*-*null*^38^ mice were generated as described elsewhere. All animals were housed in a temperature-controlled (24°C) facility with 12-h light/dark cycles (08:00 to 20:00 light) and allowed free access to water and NCD (CE-2; CLEA Japan) or HFD described in Supplementary Table 1. Food intake and body weights were monitored twice a week and core body temperature was measured using a rectal thermometer probe at 13:00. All mice were sacrificed at 13:00 and blood was taken from inferior vena cava. Serum TG, cholesterol, NEFA, and glucose levels were determined by Triglyceride E-Test Wako, Cholesterol E-Test Wako, NEFA C-Test Wako, Glucose C-II Test Wako (Wako Pure Chemical Industries), respectively. Serum insulin, leptin and adiponectin levels were determined by ELISA using an immunoassay kit (Shibayagi, Gunma, Japan). For bone marrow transplantation, total bone marrow hematopoietic progenitor donor cells harvested from *Cd300a−/−* or wild-type mice on CBL/6JJcl (C57BL6/J) background (backcrossed into C57BL6/J for 8 generations) were transplanted via orbital vein injection into lethally irradiated wild-type or *Cd300a−/−* mice, respectively, on C57BL6/J background (1100 rads; Cobalt-60 source) with a minimum cell dose of 10^6^ mononuclear cells per mouse. Transplanted mice were housed in microisolator cages for 4 weeks prior to challenge with HFD. All data are presented as mean ± s.e.m. All mouse protocols were approved by the Animal Care and Use Committee of the University of Tokyo and Tsukuba.

### Resting metabolic rate measurement

Seven to eleven weeks after HFD feeding was started, oxygen consumption was measured using open circuit indirect calorimetry (Model MK-5000, Muromachikikai, Tokyo). The chamber volume was 720 ml, airflow to the chamber was 500 ml/min, samples were taken every 3 min and a standard gas reference was taken every 30 min. Mice were kept in the metabolic chamber and acclimated for 1 day.

### Acute fat loading test

Acute fat loading test were performed in 12 week old HFD fed *Cd300a+/+* and *Cd300a−/−* mice. Mice were orally administered 3 ml/kg BW of olive oil (Sigma, O1514) and blood samples were drawn from the tail vein at 0, 1, 2, 4, and 6 h after administration. Serum triglyceride and NEFA were measured.

### HPLC analysis

An improved high resolution HPLC analysis of plasma lipoprotein was performed as described previously^39^. 20 μl of plasma was mixed with 180 μl of saline and applied to four columns of TSK Gel Lipopack XL (Tosoh, Tokyo) connected in tandem. The detection of cholesterol and triglycerides in the post-column effluent was conducted by a simultaneous profiling system for lipoprotein cholesterol, triglyceride, and free glycerol in an on-line system.

### Statistical analyses

All data are presented as mean ± s.e.m. The homogeneity in variance was evaluated by Bartlett test followed by parametric or non-parametric Dunnett**'**s multiple comparison test (one-side). The Student**'**s or Aspin-Welch *t*-test (one-side) was used to compare the data between the control and treated groups. *P < 0.05, **P < 0.01.

## Author Contributions

T.T., T.K. and J.S. planned the majority of experiments, and T.T. and J.S. wrote the paper. T.T. executed most of the experiments and K.I. assisted with the animal treatments and qPCR experiments. A.S., S.T.H. and T.N. provided CD300a−/− mice and performed the bone marrow transplantation. M.N. and S.J. performed the histological experiments. H.A. and S.T. contributed the ChIP-sequencing data analysis. T.I. and K.M. assisted with the animal dissection. F.J.G. provided PPARβ/δ null mice, and H.T., T.H., T.D., A.N., Y.W., T.M., M.A., K.T., K.Y., Y.T., S.R. and J.A. read and commented on the manuscript.

## Supplementary Material

Supplementary Information Supplementary Information
