# Supplementary material for: PPARβ/δ activation of CD300a controls intestinal immunity
Source: Sci Rep. 2014 Jun 24;4:5412. doi: 10.1038/srep05412 (PMC4067692; doi:10.1038/srep05412)
Supplement: Supplementary Information [file srep05412-s1.pdf]

Supplementary information

PPARβ/δ activation of CD300a controls intestinal immunity

Toshiya Tanaka, Satoko Tahara-Hanaoka, Tsukasa Nabekura, Kaori Ikeda, Shuying Jiang, Shuichi Tsutsumi, Takeshi Inagaki, Kenta Magoori, Takuma Higurashi, Hirokazu Takahashi, Keisuke Tachibana,Yuya Tsurutani, Sana Raza, Motonobu Anai, Takashi Minami, Youichiro Wada, Koutaro Yokote, Takefumi Doi, Takao Hamakubo, Auwerx Johan, Gonzalez Frank J., Atsushi Nakajima, Hiroyuki Aburatani, Makoto Naito, Akira Shibuya, Tatsuhiko Kodama & Juro Sakai.

Supplementary Method

Haematoxylin and eosin (H&E) staining for paraffin sections. Three micron-thick sections of the small intestine from the paraffin-embedded specimen were prepared for routine hematoxylin-eosin staining. Sections were deparaffinized in xylene for 5 min 3 times, and 5 min in ethanol 3 times. Slides were washed 3 times with water and stained with Mayer's hematoxylin for 15 minutes. After washing for 15 minutes in water, slides were counterstained in Eosin 15 minutes and washed 3 times with water. Slides were dehydrated in ethanol solutions of increasing concentration. Finally, slides were washed three times in xylene for 3 min and mounted with mounting medium.

Haematoxylin and eosin (H&E) staining for frozen sections. The small intestines and fats were fixed for 4 hours at 4°C in periodate-lysine-paraformaldehyde (PLP), washed for 4 hours in three changes of PBS containing 10%, 15% and 20% sucrose, then for 1 hour in PBS containing 20% sucrose + 5% glycerin and embedded in OCT compound (SAKURA, Tissue-Tek). Tissue specimens were cut into 6 μm thick sections using a cryostat (LEICA, CM1850). Frozen sections were processed following routine hematoxylin-eosin (H&E) staining procedures. Briefly, 6 μm frozen sections were fixed in periodate-lysine-paraformaldehyde (PLP), and washed in water. Nuclear staining was done by immersing sections in Mayer's hematoxylin for 1 minute. Sections were washed in water for 15 min, and immersed in Eosin for 1 min, and again washed in water. Slides were dehydrated in ethanol solutions of increasing concentration. Finally, slides were washed three times for 3 min in xylene and mounted with mounting medium.

Immunohistochemistry for paraffin sections. Three micron-thick paraffin sections of small intestine and pancreas were prepared for routine immunohistochemical procedures. Sections were deparaffinized in xylene and in ethanol 3 times for 5 min . For immunohistochemistry, activation of Podoplanin was performed in 10 mM Tris-1 mM EDTA (pH9.0), activation of F4/80 was performed by trypsin but no activation was done for insulin. Antigen retrieval was done by autoclave at 121°C for 15 min. Then, the glass slide chamber was removed, and allowed to come to room temperature (40 minutes). Slides were washed 3 times with PBS in the slide chamber. Endogenous peroxidase activity was blocked by freshly prepared 0.3% H_2O_2 in methanol for 20-30 minutes. Slides were washed three times for five minutes with PBS and blocked with normal serum for 10 min. Blocked slides were incubated with the primary antibody, F4/80 (AbD), Podoplanin (Santa Cruz) and Insulin (Santa Cruz) overnight at 4°C,

washed with PBS three times for 5 min, and incubated with the secondary antibody. The secondary antibody for F4/80, Podoplanin, and Insulin, was Simple Stain mouse MAX-PO (Rat), Simple Stain mouse MAX-PO (G), and Simple Stain MAX-PO (Mu) (NICHIREI BIOSCIENCE INC.), respectively. Secondary antibodies were incubated for 60 min at room temperature. Slides were washed with PBS three times for five minutes. Then, slides were immersed in 3,3ô-diaminobenzidine (DAB) solution (pre-filtered 0.01% DAB in 0.05 M Tris-HCI, pH 7.4) for 10 min at room temperature. H_2O_2 was added to a final concentration of 0.01%. Slides were washed with running tap water for 3 min, counterstained with Mayer's hematoxylin for 0.5 min, and washed again with running tap water. Slides were dehydrated using increasing concentration of ethanol (85%, 95%, 100%, and 100%) for 3 min. Finally, slides were washed three times for 3 min with xylene and mounted with mounting medium.

Immunohistochemistry for frozen sections. Small intestine, liver, and colon were fixed for 4 hours at 4°C in periodate-lysine- paraformaldehyde (PLP), washed for 6 hours in three changes of PBS solutions containing 10%, 15%, 20% sucrose, then for 1 hour in PBS containing 20% sucrose + 5% glycerin and embedded in OCT compound (SAKURA, Tissue-Tek). Tissue specimens were cut into 6-εm-thick sections by cryostat (LEICA, CM1850). Slides were washed 3 times in PBS in the slide chamber. Endogenous peroxidase activity was blocked using freshly made 0.3% H_2O_2 in methanol for 20-30 minutes. Slides were washed three times for five minutes with PBS and blocked with normal serum for 10 min. We performed immunohistochemistry using the antibodies for B220 (AbD), Thy1.2 (BD) and FA/11 (AbD). Antibodies were incubated overnight at 4°C, washed three times for 5 min with PBS, and incubated with the secondary antibody. The secondary antibody used for B220, Thy1.2 and FA/11 was ECL anti rat IgG horseradish peroxidase linked species-specific F(abô)2 fragment from goat (GE Healthcare). Slides were incubated with the secondary antibody for 60 min at room temperature and washed three times for five minutes with PBS. Then, slides were developed in 3,3ô-diaminobenzidine (DAB) solution (pre-filtered 0.01% DAB in 0.05 M Tris-HCI, pH 7.4) for 10 min at room temperature. H_2O_2 was added to a final concentration of 0.01%. Slides were washed in running tap water for 3 min, then counterstained with methylene green for 10 min. Slides were dehydrated with ethanol solutions of increasing concentration (85%, 95%, 100%, and 100%) for 3 min per wash. Finally, slides were washed in xylene three times for 3 min and mounted with mounting medium.

Microscope and PC software for photograph. Nikon ECLIPS Ci and NIS-ElementsD software were used for microscopic analysis.

Cell fractionation and immunoblot analysis. The isolation and immunoblot analysis of the nuclear extracts were performed as described previously[1,2]. To prepare the nuclear extracts, THP-1 or PMA-treated THP-1 macrophages were washed with PBS and allowed to swell at 4°C for 30 min in buffer A (10 mM HEPES-KOH (pH 7.6), 1.5 mM $MgCl_2$, 10 mM KCl, 1 mM EDTA, 1 mM EGTA, 1 mM DTT, 2 μg/ml Aprotinin, 2 μg/ml Pepstatin A, 2 μg/ml Leupeptin, 8.7 μg/ml PMSF) and then passed through a 22.5 gauge needle 15 times and centrifuged at 1,000 g at 4°C for 5 min. The pellet was resuspended in buffer C (20 mM HEPES-KOH (pH 7.6), 2.5% glycerol, 420 mM NaCl, 1.5 mM $MgCl_2$, 1 mM EDTA, 1 mM EGTA, 1 mM DTT, 2 μg/ml Aprotinin, 2 μg/ml Pepstatin A, 2 μg/ml Leupeptin, 8.7 μg/ml PMSF), and the suspension was rotated at 4°C for 30 min then centrifuged at 20,000 g at 4°C for 10 min. The supernatant was used as the nuclear extract. Nuclear extracts were resolved by SDS-polyacrylamide gel electrophoresis (10%) and electroblotted to ProBlott membranes (Applied Biosystems). Membranes were blocked with Block Ace (DS Pharma Biomedical) for 20 h at 4°C. The blot was probed with antibodies and then incubated with anti IgG horse-radish peroxidase-conjugated antibodies (Sigma). Proteins were detected using SuperSignal West Dura Extended Duration Substrate (Pierce).

Transcriptome microarray analysis. For genome-wide transcription analysis, GeneChip Human Genome U133 Plus 2.0 Array or GeneChip Mouse Genome 430 2.0 array were used as described previously[1,2]. Briefly, total RNA was extracted with ISOGEN (Nippon Gene Inc) from THP-1 macrophages, peritoneal macrophages, and small intestine. After *in vitro* transcription (IVT) and cRNA fragmentation, fragmented IVT product was hybridized on array and stained with streptavidin phycoerythrin using the manufacturerÕs recommended protocol. The arrays were scanned using the Affymetrix GeneChip Scanner 3000 (Affymetrix). To calculate the average differences for each gene probe, the GeneChip Analysis Suite software version 5.0 was used.

Chromatin immunoprecipitation (ChIP). 100 million THP-1 macrophages were cross-linked with 1% formaldehyde for 10 min at room temperature. After neutralization by 5 min incubation with 200 mM glycine, cells were collected, re-suspended in 2 ml of SDS lysis buffer (10 mM Tris-HCl (pH8.0), 150 mM NaCl, 1%

SDS, 1mM EDTA, and Protease inhibitor cocktail (Sigma)), and fragmented by the sonication (Sonifier 250, Branson; 10 min, 60% duty, output level 4). The sonicated solution was diluted with ChIP dilution buffer (20 mM Tris-HCl (pH 8.0), 150 mM NaCl, 1 mM EDTA, and 1% Triton X-100) to 10 ml final volume. Immunoprecipitaion was performed using antibodies against H3K4me3 (Abcam, ab8580), H3K4me1 (kindly gifted by Dr. Kimura), CTCF (Millipore, 07-729), PPARβ/δ (Y-9705), RXRα (K8508), and Dynabeads Protein G (Dynal/Invitrogen) for 20 h at 4°C. Immunoprecipitated samples was washed twice with ChIP dilution buffer, once with medium salt wash buffer (20 mM Tris-HCl (pH8.0), 250 mM NaCl, 0.1% SDS, 1% Triton X-100, 2 mM EDTA, and 1 mM PMSF), once with LiCl buffer (10 mM Tris-HCl (pH8.0), 250 mM LiCl, 0.5% NP-40, 0.5% sodium deoxycholate, 1 mM EDTA, and 1 mM PMSF), and 3 times with TE, respectively. To elute the protein-DNA complex from the antibody, 300 μl of elution buffer (50 mM Tris-HCl (pH8.0), 10 mM EDTA, and 1% SDS) was added to each samples and crosslinks were reversed by heating at 65°C for 20 h. After reverse crosslinking, 100 μg RNAse A was added and samples were incubated for 45 min at 37°C. These samples were treated with 60 μg Proteinase K at 55°C for 1.5 h. The immunoprecipitated DNA was extracted with phenol-chloroform-isoamylalcohol. The aqueous phase was transferred to a 1.5 ml tube and ethanol-precipitated in the presence of 20 μg glycogen. Precipitated DNA was dissolved in EB buffer and purified using Qiaquick PCR purification kit (Qiagen). Prepared DNA was quantified using Q-bit (Invitrogen) and more than 10 ng of DNA was processed for qPCR or sequence.

ChIP-seq. ChIP-DNA or input control was prepared for sequencing according to the Illumina/Solexa Genomic DNA protocol. Fragmented DNA was ligated with Solexa linkers. A 1:100 dilution of the Adaptor Oligo Mix (Illumina) was used in the ligation step. Subsequently, PCR was performed with 18 cycles, and samples were subjected to gel electrophoresis in 2% agarose. A narrow range of fragment sizes, between 175-225 bp, was excised and the containing DNA was eluted. The generated DNA library (2pM) was applied to the flow-cell using the Cluster Station device from Illumina. The ʻpoloniesȏ generation and the sequence-readings were performed with Illumina Genome Analyzer 1G, according to the protocol from Illunima. The obtained sequences were mapped onto human genomic sequences (hg18 as of UCSC Genome Browser) using the sequence alignment program Eland (Illumina) allowing no more than two mismatches per sequence[3]. For visualization, we used IGB (integrated genome browser, Affymetrix) software. MEME (Multiple Em for Motif Elicitation)

program version 4.6.0 was used for searching the motifs enriched by PPARβ/δ-RXRα-binding in THP-1 macrophages.

Quantitative real-time PCR (qPCR). The qPCR method has been described[7]. Specific primers for each gene were designed using the Primer Express software (Life Technologies). All primer sequences used in this paper are available on request. Total RNA was isolated using an RNA preparation kit (Isogen; Nippon Gene Corp.). First strand cDNA was synthesized from total RNA with oligo dT primers using SuperScript II reverse transcriptase (Life Technologies). The real time PCR mixture contained, in a final volume of 20 εL, 20 ng of reverse transcribed total RNA (5 εL of cDNA solution), 167 nM of forward and reverse primers, and 10 εL of 2 x SYBR Green PCR Master Mix (catalog no. 4312704; Life Technologies). PCR was carried out in 384-well plates using the ABI PRISM 7900HT Sequence Detection System (Life Technologies). All reactions were performed in triplicate. The relative amount of all mRNAs was calculated using the comparative CT method. Cyclophilin mRNA was used as the invariant control for all studies.

Glucose tolerance test (GTT). Oral glucose tolerance test (GTT, 2 g/kg glucose) were performed in *Cd300a+/+* and *Cd300a-/-* mice fed NCD or HFD for 9 weeks. Blood samples were drawn from the tail vein at 0, 15, 30, 60 and 120 min and assayed for serum glucose and insulin.

References

1. Tanaka T*, et al.* Activation of peroxisome proliferator-activated receptor delta induces fatty acid beta-oxidation in skeletal muscle and attenuates metabolic syndrome. *Proc Natl Acad Sci U S A* **100**, 15924-15929 (2003).

2. Wakabayashi K*, et al.* The peroxisome proliferator-activated receptor gamma/retinoid X receptor alpha heterodimer targets the histone modification enzyme PR-Set7/Setd8 gene and regulates adipogenesis through a positive feedback loop. *Mol Cell Biol* **29**, 3544-3555 (2009).

3. Kanki Y*, et al.* Epigenetically coordinated GATA2 binding is necessary for endothelium-specific endomucin expression. *EMBO J* **30**, 2582-2595 (2011).

Tanaka T et al. Supplementary Figure S1

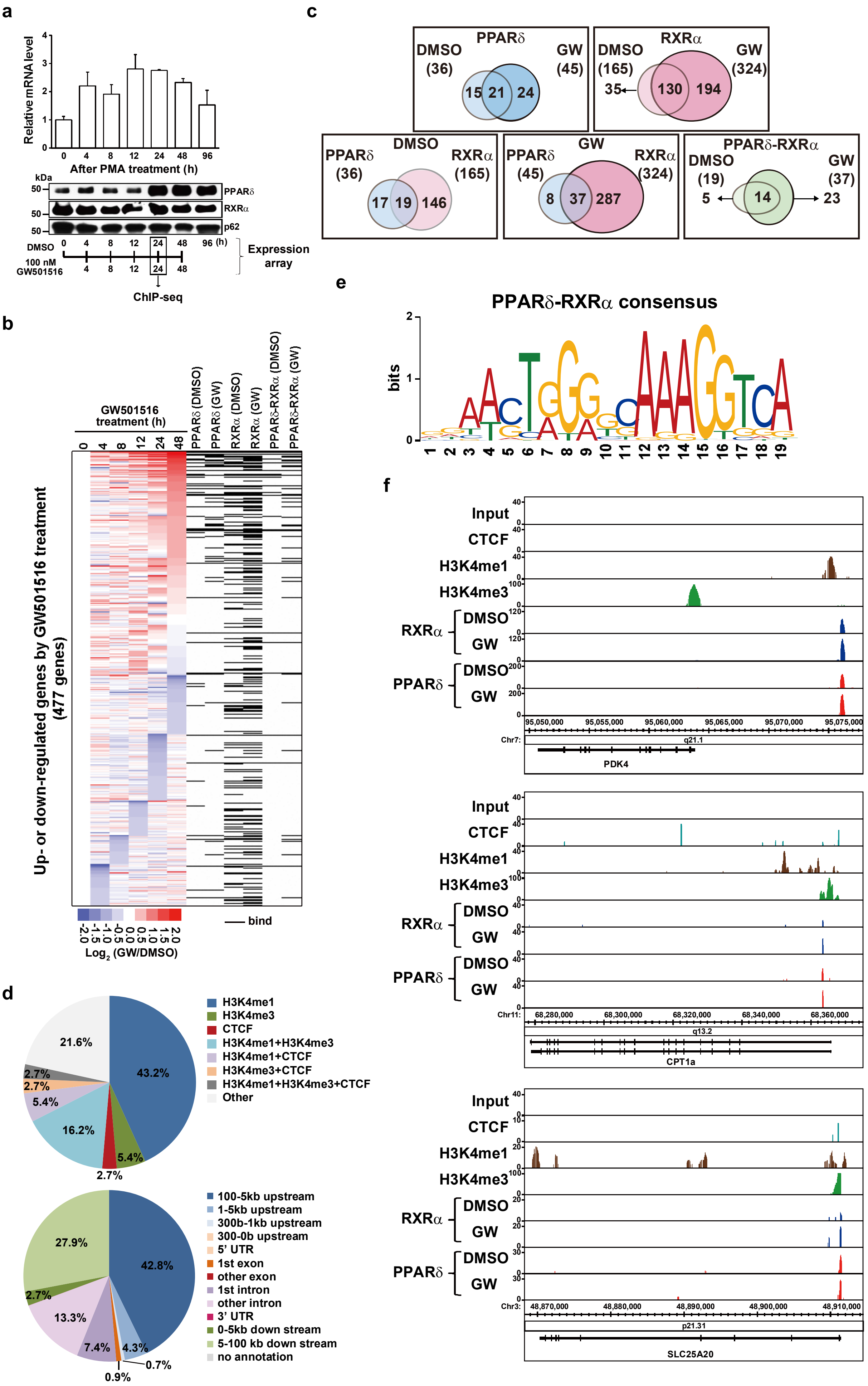

Supplementary Figure S1. Transcriptome and PPARβ/δ ChIP seq analyses in THP-1 macrophages by GW501516 treatment. (**a**) Experimental design: THP-1 macrophages were cultured and treated with either GW501516 (100 nM) or vehicle control (DMSO) and harvested at the indicated times. ChIP seq analyses for PPARβ/δ, RXRα, H3K4me1, H3K4me3, and CTCF were performed 24 h after treatment and gene expression profiling was performed at 0, 4, 8, 12, 24, 48 h using an Affymetrix Human Genome U133 Plus 2.0 Array. PPARδ mRNA levels and the immuoblots for PPARβ/δ, RXRα, and p62 are shown. mRNA levels are shown as the mean ± s.e.m. relative to cyclophilin of three samples analyzed in triplicate. (**b**) Heat map showing expression changes of genes in THP-1 macrophages associated with GW501516 treatment. (**c**) Venn diagrams illustrating the overlap of either PPARβ/δ or RXRα binding sites in the absence or presence of GW501516. (**d**) Overlap between ChIP-seq peaks of PPARβ/δ-RXRα and CTCF bound, H3K4me1, and H3K4me3 marked genes (top). Location of PPARβ/δ binding peaks relative to known genes (bottom). (**e**) Identification of enriched motifs of PPARβ/δ and RXRα binding sequences in THP-1 macrophages. The height of each letter represents the relative frequency of nucleotides at different positions in the consensus. (**f**) Examples of ChIP-seq on representative PPARβ/δ target genes. Vertical axes show the signal score, which reflects the fold-enrichment of the ChIP-seq samples.

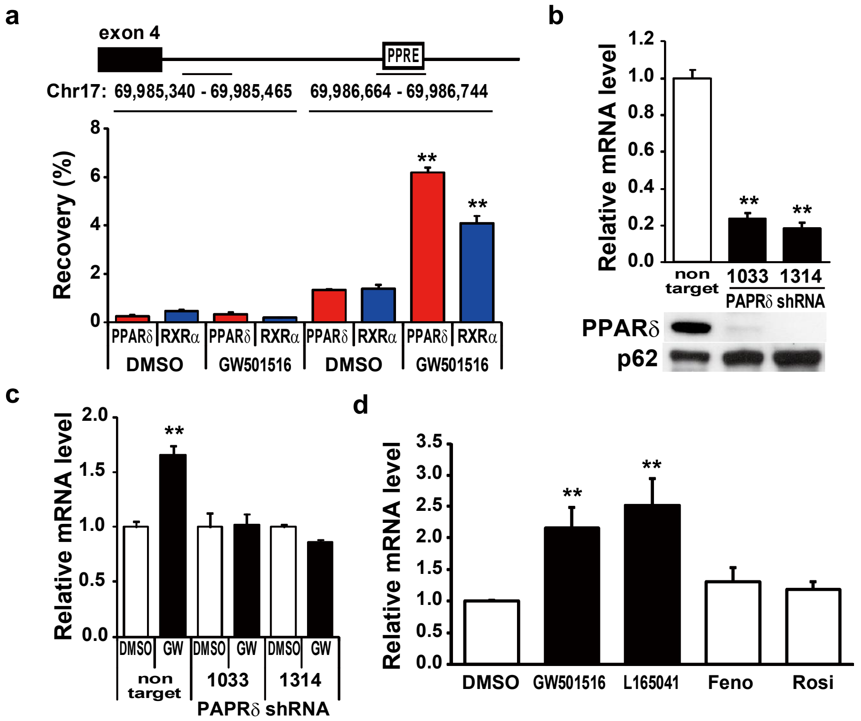

Supplementary Figure S2. PPARβ/δ directly regulates *Cd300a* gene expression through changes in histone modifications close to PPARβ/δ binding sites. (**a**) ChIP-qPCR. ChIP samples from Fig 1 were also analyzed by qPCR. All data represent recovery, in percent, of each DNA fragment relative to total input DNA. Schematic diagrams of the *Cd300a* gene above the panels indicate the DNA fragments that were amplified by qPCR following ChIP. Shown is the mean of three replicates; error bars represent s.e.m. (**b**) qPCR analysis showing that PPARβ/δ transcripts are efficiently knocked down in THP-1 macrophages by transduction of a lenti-virus carrying two different shRNAs specific to PPARβ/δ. (**c**) *Cd300a* induction by GW501516 is blunted in PPARδ knock-down THP-1 cells. THP-1 cells were cultured for 24 h with the indicated concentrations of GW501516. (**d**) The effect of various PPAR agonists on *Cd300a* expression in THP-1 macrophages. THP-1 macrophages were treated with DMSO (control), fenofibric acid (Feno) (100 μM), GW501516 (100 nM), or rosiglitazone (Rosi) (1 μM) for 24 h. Data shown are values relative to cyclophilin (± s.e.m.) of three samples analyzed in triplicate. *$P < 0.05$, **$P < 0.01$.

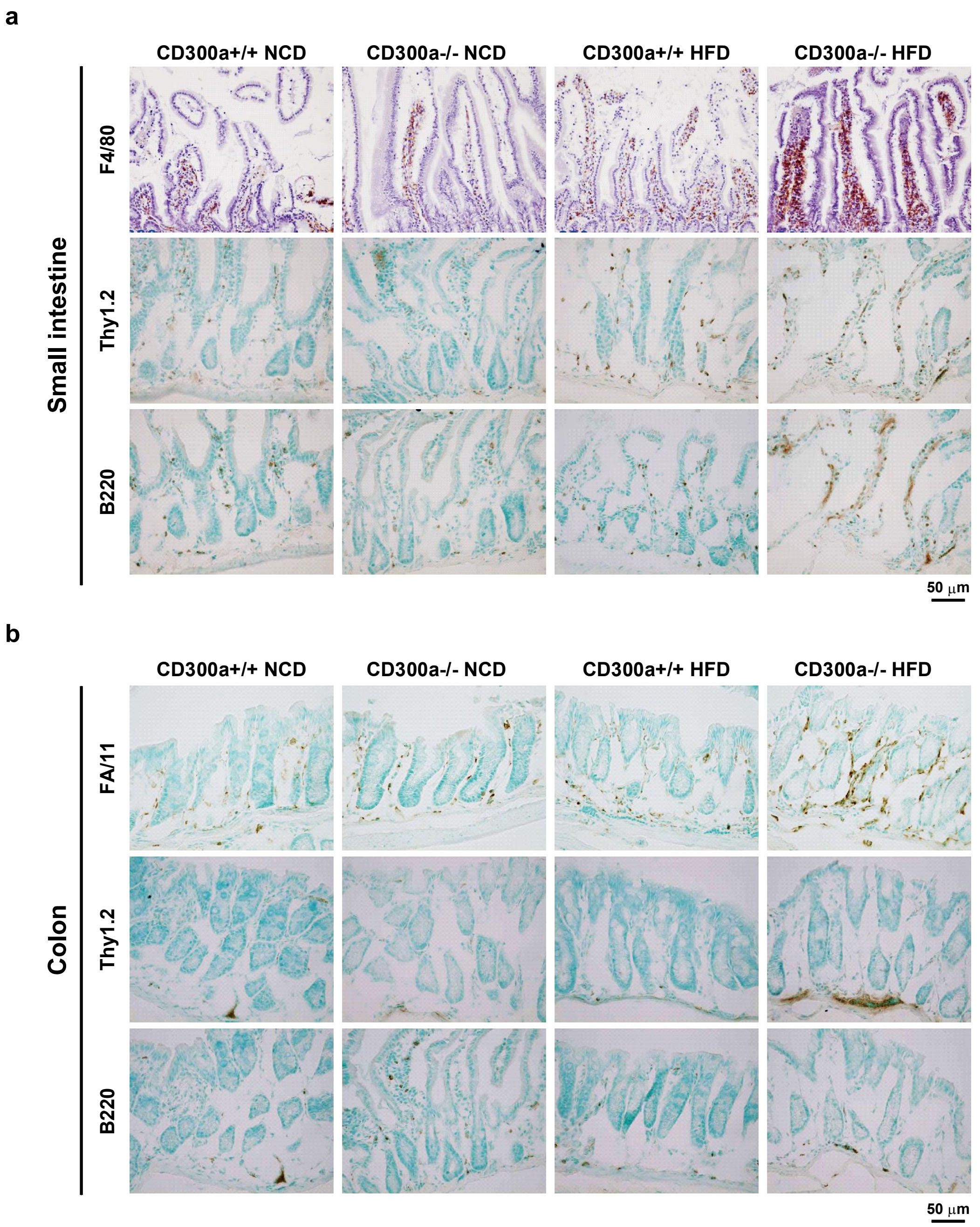

Supplementary Figure S3. Intraepithelial macrophage infiltration in lamina propria in the intestine of *Cd300a-/-* mice fed HFD. (**a**,**b**) Immunostaining of jejunal cross sections (**a**) and colon sections (**b**) from *Cd300a+/+* and *Cd300a-/-*mice (18-weeks old) on NCD or HFD used in Fig. 2. Enhanced infiltrates of T- and B- lymphocytes and macrophages were observed.

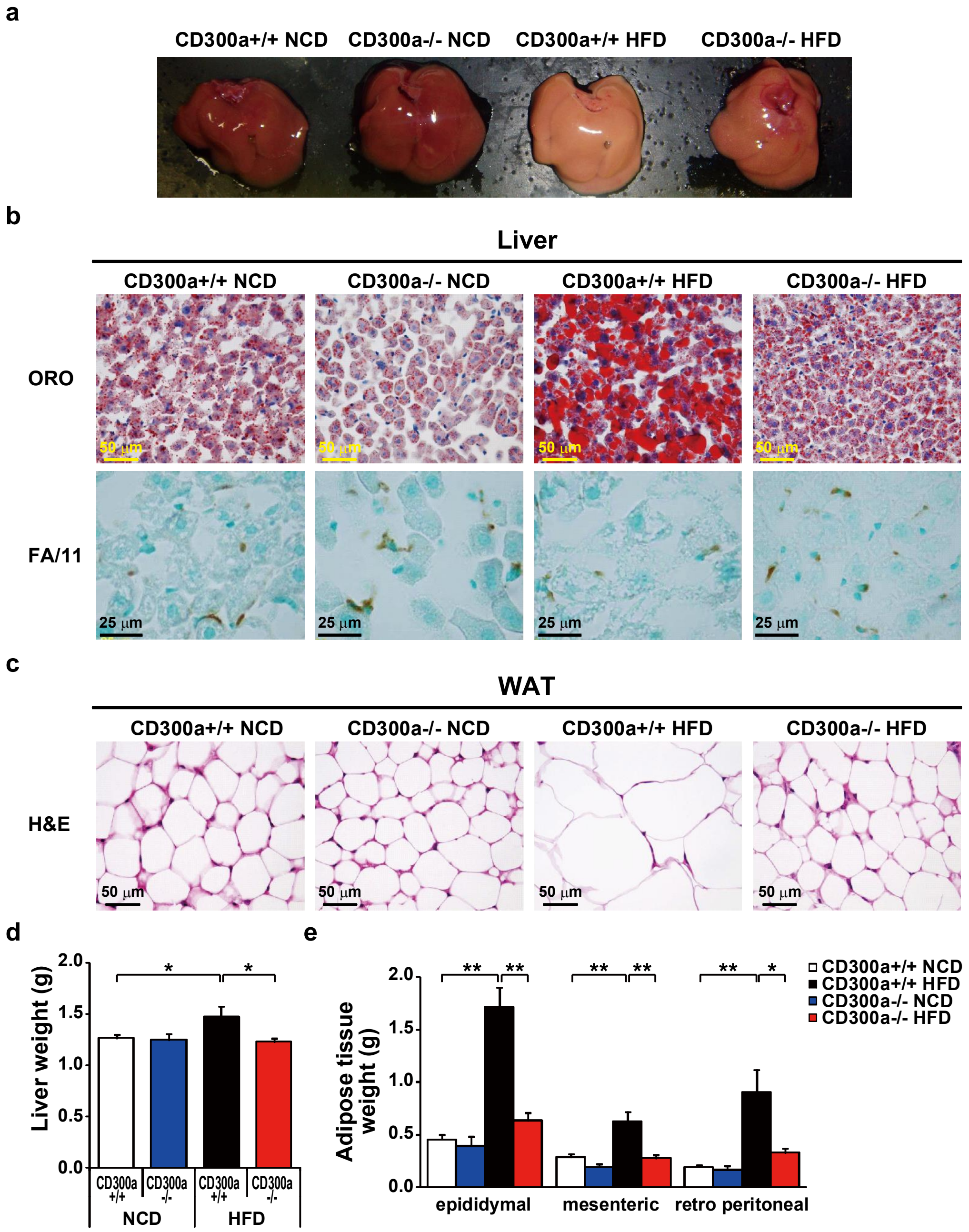

Supplementary Figure S4. Reduced fat depots in liver and white adipose tissue (WAT) in *Cd300a-/-* mice on HFD. (**a**) The gross morphology of the liver of *Cd300a+/+* and *Cd300a-/-* mice fed NCD or HFD for 14 weeks used in Fig. 2. (**b**) ORO staining and CD68 (FA/11) immunostaining of liver. (**c**) H&E staining of epididymal fat (WAT) of *Cd300a+/+* and *Cd300a-/-* mice on NCD or HFD for 14 weeks. (**d,e**) Weights of liver (**d**) and retroperitoneal, mesenteric, and epididymal fat (**e**) after 14 weeks of NCD or HFD (n = 5-6 per group). Data represent ± s.e.m. $*P < 0.05$; $**P < 0.01$.

Tanaka T et al. Supplementary Figure S5

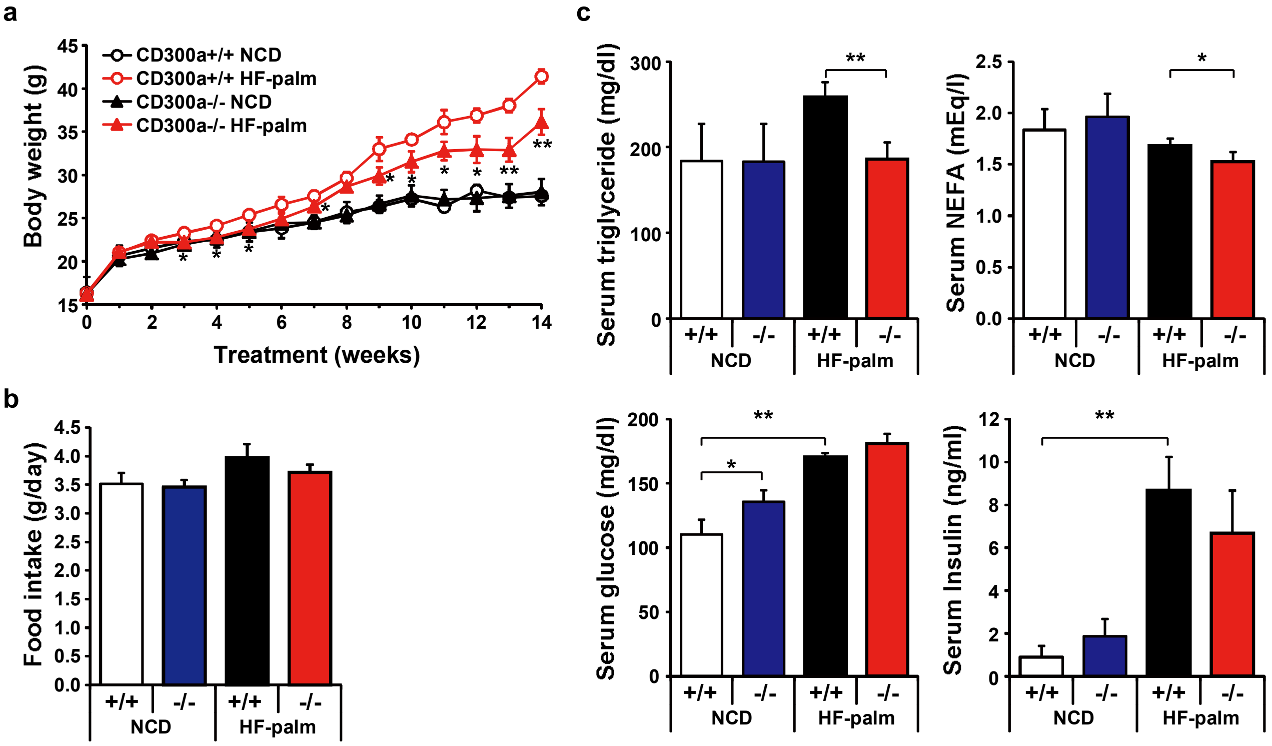

Supplementary Figure S5. Palm oil-based saturated fatty acids (sFAs) also reduced body weight gain in *Cd300a-/-* mice. (**a**) Growth curves of *Cd300a*+/+ and -/- mice on NCD (CE-2), or palm oil-based HFD (HF-palm, D07081501). (**b**) Food intake. (**c**) Metabolic parameters. Data represent the mean ± s.e.m for 4 animals per group. $^{*}P < 0.05$, $^{**}P < 0.01$ compared with *Cd300a*+/+ mice on the same diet.

Tanaka T et al. Supplementary Fig S6

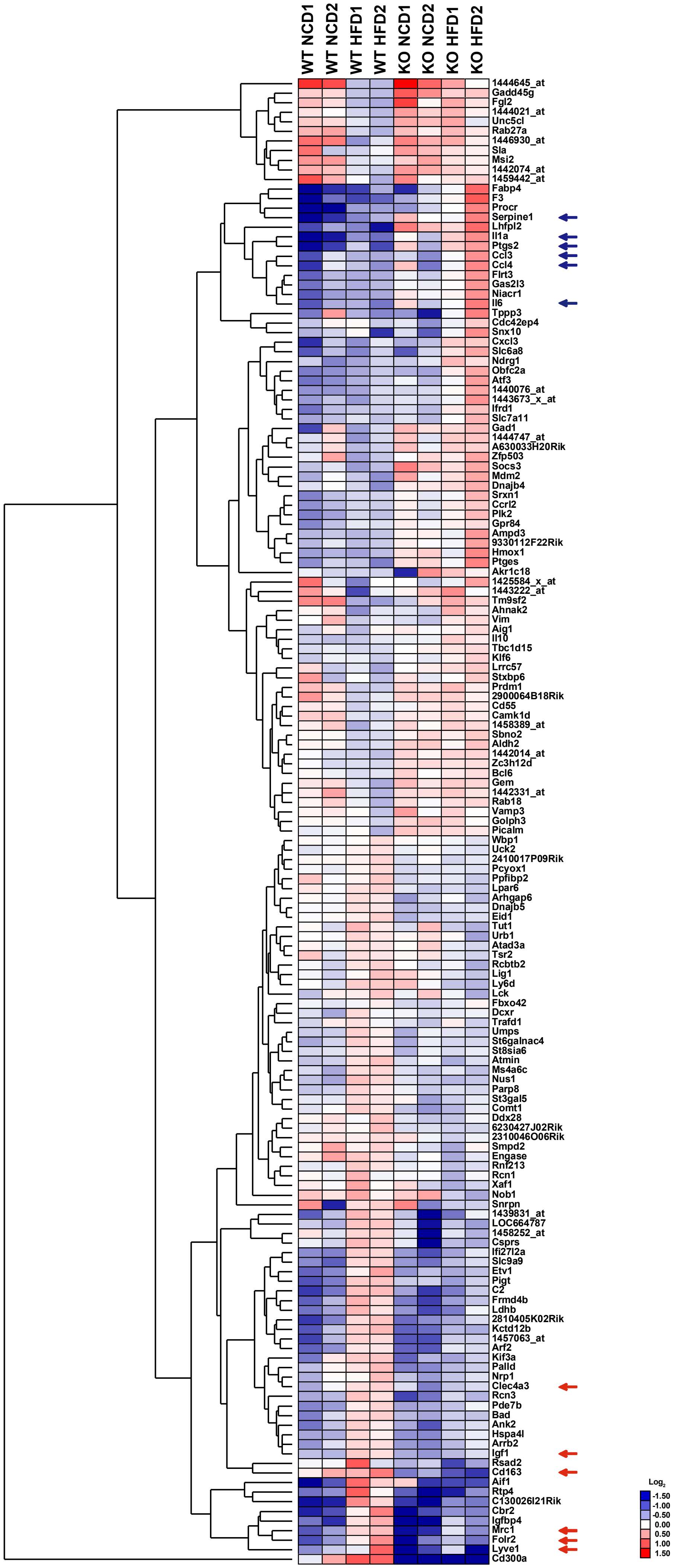

Supplementary Figure S6. Cluster analysis of peritoneal macrophage gene expression in response to high-fat feeding. *Cd300a-/-* and *Cd300a+/+* littermates (from Fig. 2) were sacrificed for peritoneal macrophage gene expression analyses. Shown are genes whose transcript abundance is increased more than 1.23-fold or decreased greater than 1.23-fold in *Cd300a-/-* mice in response to high fat feeding as compared to their *Cd300a+/+* littermates on HFD. For reference, a color intensity scale is included at the bottom. Blue and red arrows denote representative M1 and M2 markers, respectively.

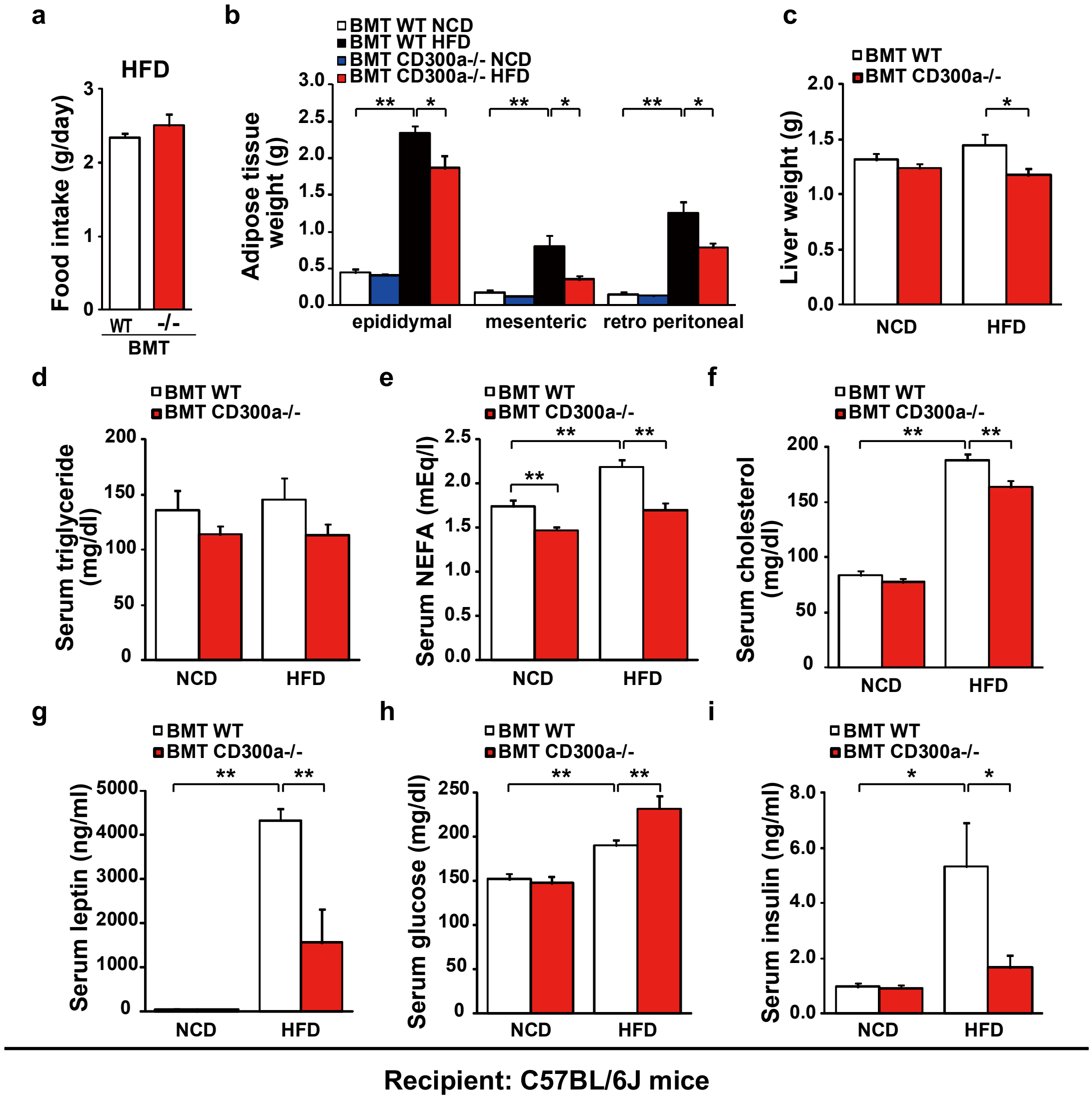

Supplementary Figure S7. Reduced lipid accumulation in *Cd300a-/-* BMT mice on HFD. (**a**) Food intake. (**b-i**) Weights of various adipose tissues (**b**), liver weights (**c**), and metabolic parameters (**d-i**) of BMT *Cd300a-/-* or *Cd300a+/+* mice (C57BL/6J background) on NCD or HFD used in Fig. 4. Data represent the mean ± s.e.m for 4-6 animals per group. *$P < 0.05$, **$P < 0.01$.

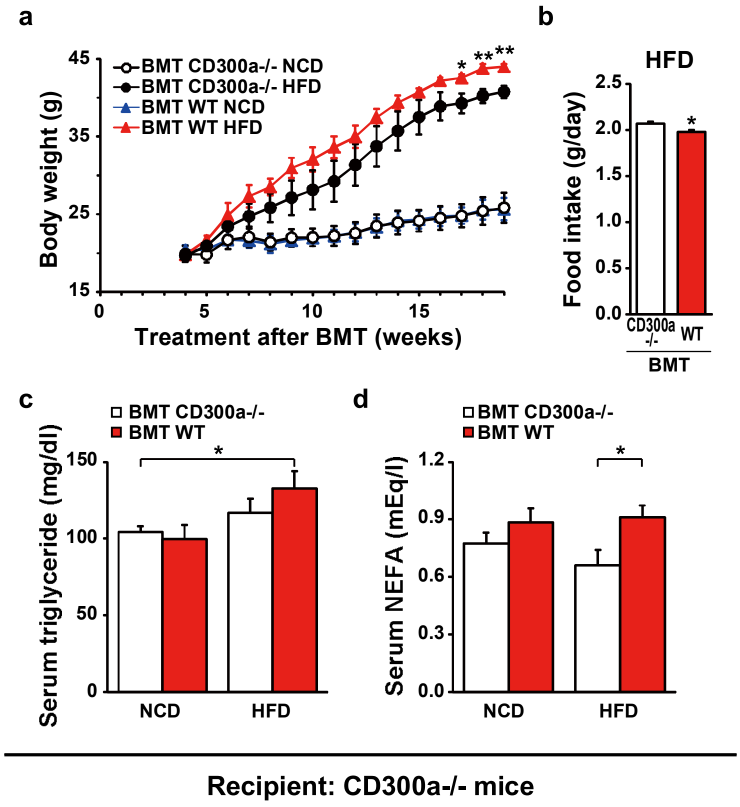

Supplementary Figure S8. ***CD300a+/+* bone marrow corrects the reduced body weight gain phenotype of *Cd300a-/-* mice on HFD.** (**a**) Body weight changes of *Cd300a-/-* (C57BL/6J background) littermates reconstituted with bone marrow of either wild-type or *Cd300a-/-* (BMT WT and BMT *Cd300a-/-* mice, respectively) on HFD or NCD. *$P < 0.05$, **$P < 0.01$ compared with BMT *Cd300a-/-* mice on HFD. (**b**) Mean daily food intake for 15 weeks. (**c**,**d**) Serum TG (**c**) and NEFA (**d**) concentrations. *$P < 0.05$. Data represent ± s.e.m for 4-5 animals per group.

Tanaka T et al. Supplementary Figure S9

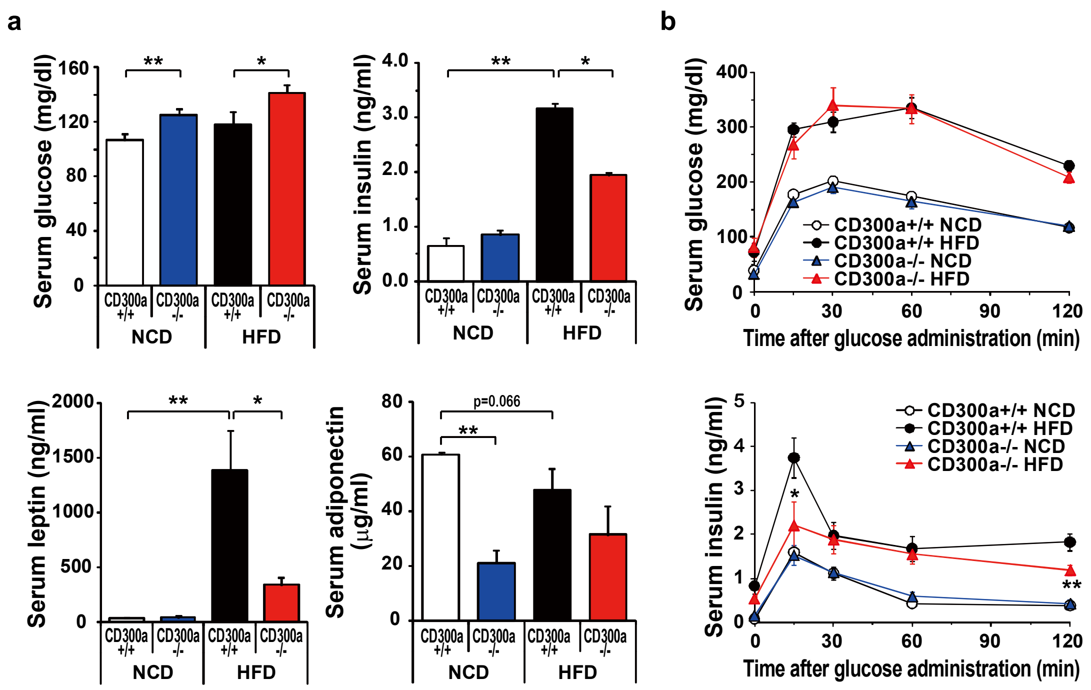

Supplementary Figure S9. Metabolic parameters of *Cd300a-/-* mice on NCD and HFD. (**a**) Metabolic parameters of 13-14-week-old mice under fed state (*Cd300a+/+*, *n* = 6; *Cd300a-/-*, n = 5). **P* < 0.05, ***P* < 0.01. (**b**) Oral glucose tolerance test (2 g/kg glucose). Serum glucose (upper panel) and insulin (lower panel) concentrations. **P* < 0.05, ***P* < 0.01 compared with *Cd300a+/+* mice on HFD. Data represent the mean ± s.e.m for 5-6 animals per group.

a

	High fat diet	
	gm(%)	*kcal*(%)
Protein	18.4	13.9
Carbohydrate	36.8	27.7
Fat	34.5	58.5
Total		100.0
kcal/gm	5.32	
**Ingredient**	**gm**	
Casein	22.00	
Dextrin	7.00	
Lard	35.50	
Maltose	30.05	
AIN-93G Mineral mix	3.50	
AIN-93 Vitamin mix	1.00	
Methionine	0.70	
Choline Bitartrate	0.25	
Total	100.00	

	(%)
C14:0, Myristic	1.1
C16:0, Palmitic	25.7
C16:1, Palmitoleic	2.5
C18:0, Stealic	15.4
C18:1, Oleic	42.7
C18:2, Linoliec	10.9
C18:3, Linolenic	1.0
C20:1	0.6
Total	100.0
Saturated(%)	42.2
Monosaturated(%)	45.8
Polyunsaturated(%)	11.9

b

**Product #**	**HF-palm**	
	**gm(%)**	***kcal*(%)**
Protein	24	20
Carbohydrate	41	35
Fat	24	45
Total		100.0
kcal/gm	4.73	
**Ingredient**	**gm**	**kcal**
Casein, 80 Mesh	200	800
L-Cysteine	3	12
Corn Starch	72.8	291
Maltodextrin 10	100.0	400
Sucrose	172.8	691
Cellurose, BW200	50	0
Soybean Oil	25.0	225
Palm Oil	177.5	1598
Mineral Mix S10026	10.0	0
DICalcium Phosphate	13.0	0
Calcium Carbonate	5.5	0
Potassium Citrate, H_2O	16.5	0
Vitamin Mix V10001	10	40
Choline Bitartrate	2	0
FD&C Yellow Dye #5	0.025	0
FD&C Blue Dye #1	0.025	0
**Total**	**858.15**	**4057**

**Product #**	**HF-palm**
**Ingredient**	
Palm Oil, RBD	177.5
Soybean Oil	25.0
**Total**	**202.5**
C12:0, Lauric	0.5
C14:0, Myristic	1.4
C16:0, Palmitic	69.5
C16:1, Palmitoleic	0.2
C18:0, Stearic	10.2
C18:1, Oleic	80.6
C18:2, Linoleic	35.9
C18:3, Linolenic	2.8
C20, Arachidic	0.9
**Total**	**202.1**
Saturated (g)	82.5
Monounsaturated (g)	80.8
Polyunsaturated (g)	38.8
Saturated (%)	40.8
Monounsaturated (%)	40.0
Polyunsaturated (%)	19.2

Supplementary Table S1. Nutrient composition of HFD and palm oil-based HFD. (**a**,**b**) Macronutrient, ingredient and fatty acid composition of lard-based HFD (Oriental Yeast, Tokyo, Japan) (**a**) and palm oil-based HFD (D07081501, Research Diets Services, Wijk bij Duurstede, Netherlands) (**b**). Fatty acid composition is expressed as percentage of total fatty acids.
